# The immediate effect of alternate rapid maxillary expansions and constrictions on the alveolus: a retrospective cone beam computed tomography study

**DOI:** 10.1186/s40510-018-0237-x

**Published:** 2018-10-15

**Authors:** Narayan H. Gandedkar, Eric Jein-Wein Liou

**Affiliations:** 1 Cleft and Craniofacial Centre and Dental Service, KK Women’s and Children’s Hospital, 100 Bukit Timah Road, Singapore, 229899 Singapore; 2 Department of Craniofacial Orthodontics, Craniofacial Research Center, Chang Gung Memorial Hospital, 6F 199 Tung-Hwa North Road, Taipei, Taiwan; 3 Graduate Institute of Craniofacial Medicine, Chang Gung University, 259 Wen-Hwa 1st Road, Kwei-Shan Tao-Yuan, Taiwan

**Keywords:** Alt-RAMEC, Expansion protocol, Anchor tooth, Buccal alveolar bone thickness, Palatal alveolar bone thickness

## Abstract

**Background:**

Rapid maxillary expansion reduced the expander’s anchor teeth buccal alveolar bone thickness. However, the effects of alternate rapid maxillary expansions and constrictions (Alt-RAMEC) on the expander’s anchor teeth alveolar thickness has not been assessed. The purpose of this retrospective study was to evaluate the effects of Alt-RAMEC on the alveolus surrounding the anchor teeth of a double-hinged expander.

**Methods:**

Twenty-six individuals, including 12 males (11.5 ± 1.00 years) and 14 females (11.5 ± 0.90 years), who had double-hinged expander for 7 weeks of Alt-RAMEC and then 3 months of maxillary protraction, were included. Their cone beam computed tomography (CBCT) images taken 3–6 months before treatment (T0) and after 7 week of Alt-RAMEC (T1), were studied for the buccal alveolar bone thickness (BABT) and palatal alveolar bone thickness (PABT) of the expander’s anchor teeth (first molars and first and second premolars) in four axial sections. The intra-class correlation coefficient, Dahlberg’s formula, and paired *t* tests were used to analyze the method errors, and the intra-group changes of the BABT and PABT at T0-T1 were analyzed by paired *t* test (*p* < 0.05).

**Results:**

The 7 weeks of Alt-RAMEC significantly reduced the BABT of the expander’s anterior anchor teeth (0.54~ 70 mm, *p* < 0.05) and at the cervical region (0.14~ 0.25 mm, *p* < 0.05), but not at the apical region of the expander’s posterior anchor teeth. The reduction of BABT by 7 weeks of Alt-RAMEC was within the scope of the initial BABT. On the opposite, the Alt-RAMEC significantly (*p* < 0.05) increased the PABT in the anterior anchor teeth and the cervical region of posterior anchor teeth.

**Conclusions:**

A 7-week protocol of Alt-RAMEC with double-hinged expander for maxillary protraction might reduce the buccal alveolar bone thickness of the expander’s anchor teeth, although the reduction is within the scope of initial alveolar thickness of the expander’s anchor teeth.

## Background

The alternate rapid maxillary expansions and constrictions (Alt-RAMEC) protocol was developed to elicit circumaxillary suture disjunction and facilitate subsequent maxillary protraction in growing skeletal class III individuals [1–3]. The rationale behind the protocol is to amplify the effects of rapid maxillary expansion (RME) by increasing the frequency of expansion through alternating rapid expansion and constriction several times [4, 5]. The extent of circumaxillary suture disjunction with a 5-week Alt-RAMEC protocol, demonstrated in a cat model, was significantly greater (94.4% vs. 64.8%) than with a 1-week RME protocol [6]. The amount of maxillary protraction has been reported to be 4–6 mm in 5 months under the Alt-RAMEC protocol [1, 3], whereas it was 1.5–3.0 mm in 10 to 12 months under the RME protocol [7–11].

Because the expander is a tooth-borne device, the immediate effects of the protocol on the dentoalveolar apparatus of the anchor teeth cannot be underemphasized. Researchers have reported buccal marginal bone loss and cortical fenestration with the RME protocol [12]. Several tomography studies have also reported the effects of RME on the dentoalveolar apparatus [13–16]. The behavior of the buccal and palatal alveolar bone with tooth- and tissue-borne expanders under the RME protocol has been studied, and significant reductions in the buccal alveolar bone with bone dehiscence were noted, along with compensatory increases in palatal alveolar bone thickness [17]. Studies have reported that the initial buccal alveolar bone thickness (BABT) of maxillary anchor teeth is critical, and BABT reduction following expansion is dependent on the initial thickness with thinnest pre-expansion BABT experiencing greatest reduction, especially buccal alveolus of first premolars [15, 18]. We assume that Alt-RAMEC’s alternating expansion and constriction protocol might also lead to BABT reduction and may increase the palatal alveolar bone thickness (PABT). However, no study has been performed to verify this assumption and quantify Alt-RAMEC’s effect on the maxillary anchor teeth alveolus by comparing with pre-expansion alveolus thickness.. Hence, we conducted this retrospective study to test the following hypotheses:

1. Alt-RAMEC decreases maxillary anchor teeth buccal alveolar bone thickness in comparison to initial alveolus thickness.
2. Alt-RAMEC increases maxillary anchor teeth palatal alveolar bone thickness in comparison to initial alveolus thickness.

Therefore, the purpose of this retrospective study was to evaluate the effect of Alt-RAMEC on the alveolus surrounding the anchor teeth of an expander and compare with pre-expansion alveolus.

## Methods

This project was approved (no.102-5509B) by the ethical committee of the hospital, and informed consents were obtained from the guardians of all patients. The sample size was determined by setting an effect size 0.8, power 0.9, and significant level 0.01for a two-tailed same-subjects paired *t* test. Twenty-six growing patients with class III malocclusion (Table 1) who sought for maxillary orthopedic protraction and orthodontic treatment were included according to the following inclusion criteria: maxillary hypoplasia with ANB ≤ 0°, SNA ≤ 78°, SNB ≥ 82°, cervical vertebrae stage 2, and early permanent dentition. Patients with absence of maxillary posterior teeth, metallic restorations on the maxillary posterior teeth, previous periodontal disease, and previous orthodontic treatment were excluded.

**Table 1:** Descriptive statistics for the participants of this research project

Sex	*n* = 26	Age (Years) (Mean ± SD)
Males	12	11.5 ± 1.00
Females	14	11.5 ± 0.90

**Fig. 1:**
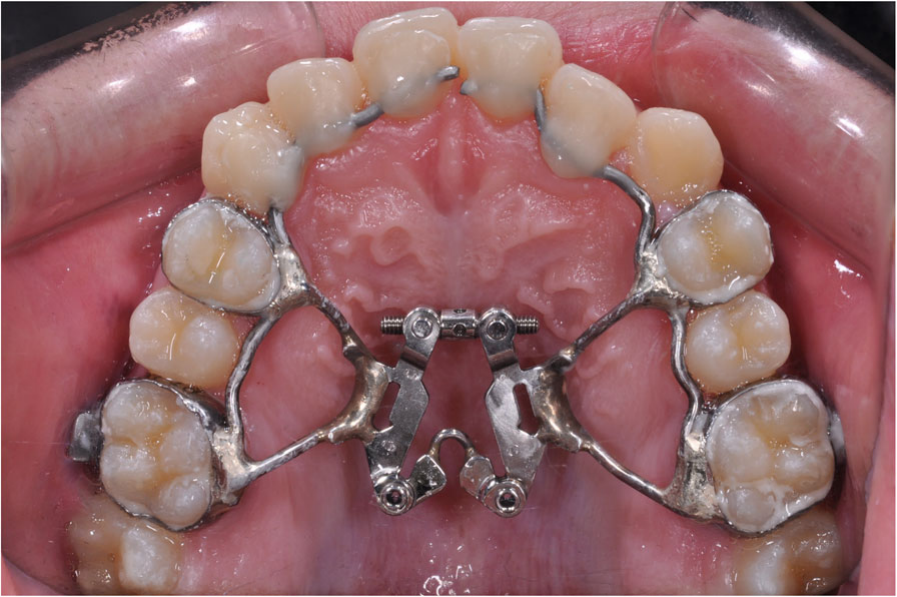
The double-hinged expander used in the study. It consists of a jackscrew with two hinges of rotation and two anterior extension arms (0.045 in. stainless steel wires)

Each patient’s maxillary first molars and first premolars were banded with a double-hinged expander (Bestdent, Kaoshiung, Taiwan) (Fig. 1.). The anterior extension arms of the expander were bonded to the palatal surface of the central, lateral incisors and canines. The expander was activated a day after the cementation. The activation protocol included 7 weeks of Alt-RAMEC, commencing with expansion in the first week, alternating to constriction in the second week, and ending with expansion in the seventh week. The daily expansion or constriction was 1.0 mm.

In this retrospective study, all patients had two sets of cone beam computed tomography (CBCT) images taken 3 to 6 months before expansion (T0), and immediately after 7 weeks of Alt-RAMEC (T1) without removing the expander. A CBCT scanner (i-CAT, Imaging Sciences International Inc., PA) was used at 120 kV and 3–8 mA, with a scanning time of 10 s and a field of view of 16 cm × 22 cm, at a 12-bit gray scale. The data of each patient were reconstructed with 0.25-mm voxel size and converted into DICOM images. The data were transferred to a network computer workstation with the Simplant Pro™ Imaging software program (Materialize, Glen Burnie, MD), in which multiplanar axial images were generated and assessed.

### Measurement of alveolar bone thickness

**Fig. 2:**
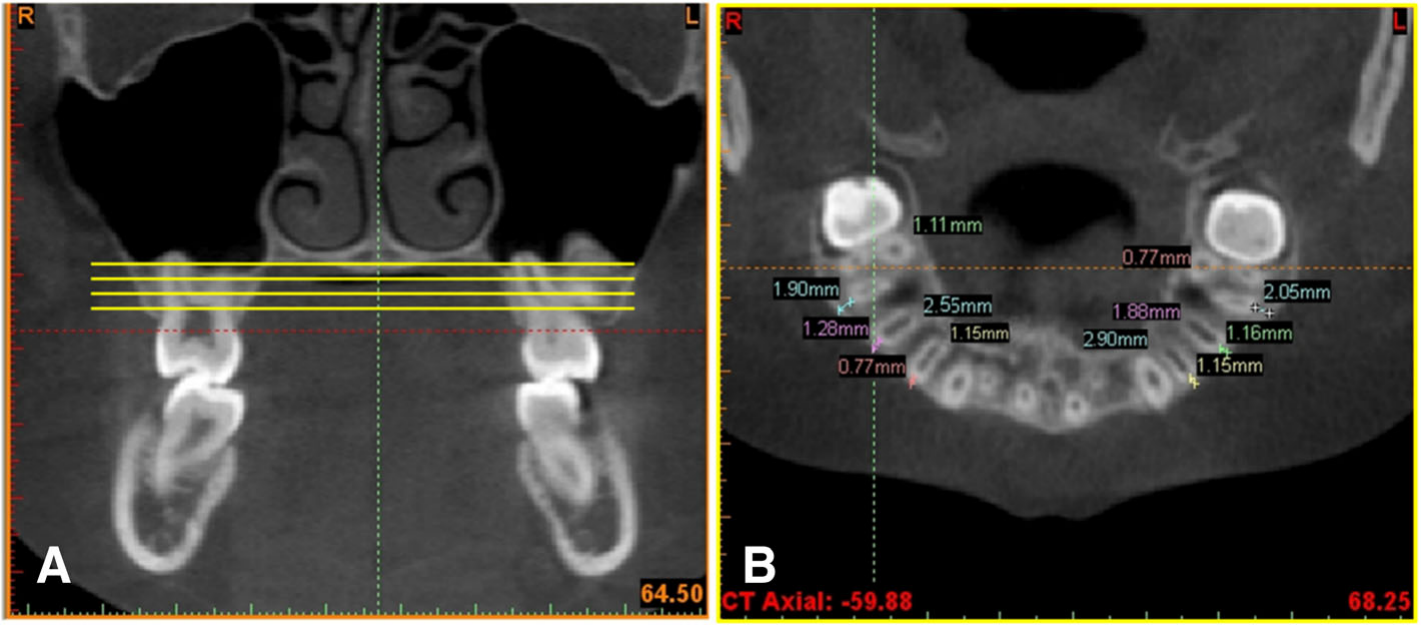
**a**. The four sections for the measurement of buccal alveolar bone thickness (BABT) and palatal alveolar bone thickness (PABT). **b**. The measurements of BABT and PABT at the axial section at the furcation of right maxillary first molar

All the patients’ CBCT images were oriented so that the palatal plane (anterior nasal spine to posterior nasal spine) in the sagittal sections and the infraorbital plane (right infraorbital foramen to left infraorbital foramen) in the coronal sections were parallel to the floor [19]. Four axial sections parallel to the palatal plane [12, 15, 16] were measured (Fig. 2).

- Axial section 1 (AS1): 1 mm cervical to the furcation of right maxillary first molar.
- Axial section 2 (AS2): at the furcation of right maxillary first molar.
- Axial section 3 (AS3): 1 mm apical to the furcation of right maxillary first molar.
- Axial section 4 (AS4): 2 mm apical to the furcation of right maxillary first molar.

The buccal alveolar bone thickness (BABT) of the maxillary posterior teeth was measured from the external border of the buccal cortical plate to the center of the buccal aspect of the first and second premolar and the mesiobuccal and distobuccal roots of the first molar. The palatal alveolar bone thickness (PABT) was measured from the external border of the palatal cortical plate to the center of the palatal aspect of the root of the first and second premolar and to the center of the palatal root of the first molar. Buccally and palatally displaced teeth with rotation were measured at the nearest root distance to the external contour of the alveolar ridge.

### Statistical analysis

All the measurements were performed by the same examiner at the same scanner console and were repeated after a month interval by the same examiner. The intra-class correlation coefficient and simple linear scatter plot were used to analyze the repeated measures, and the Dahlberg’s formula (√(ΣD^2^/2 N) [20] was used for the method errors. The reliability was tested by Cronbach’s alpha analysis. The right and left side BABT and PABT of each section of each tooth were pooled respectively, and a paired *t* test (*p* < 0.05) was used to evaluate their changes at T0–T1.

## Results

**Fig. 3:**
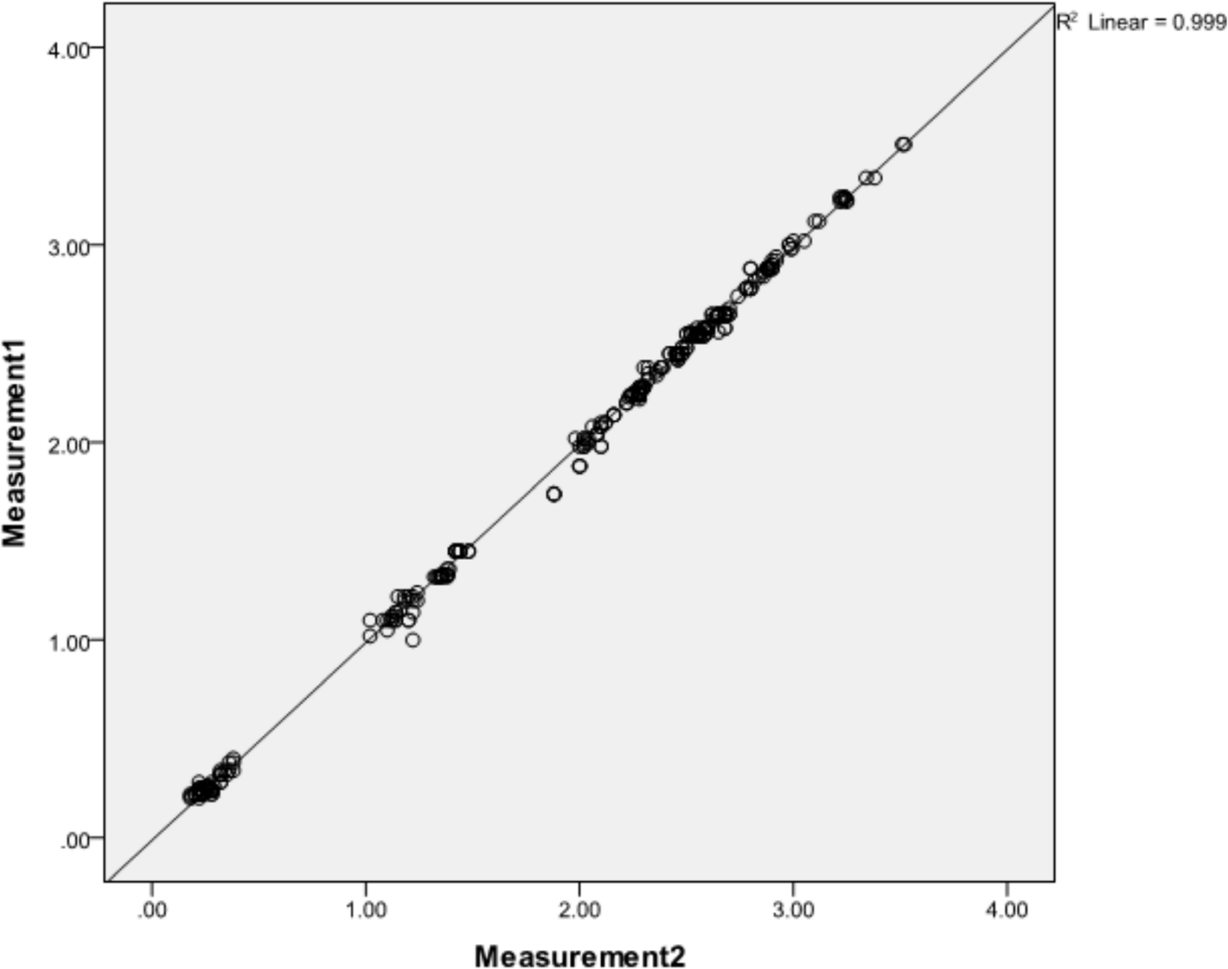
The scatter plot indicates the agreement between the first and second measurements fitting to a 45-degree slope regression line (*R*^2^ linear = 0.999)

The ICC coefficient at a 95% confidence interval varied from 0.992 to 0.996, and the simple linear scatter plot indicated excellent agreement between the first and second measurements (*R*^2^ linear = 0.999) (Fig. 3). The method errors were 0.002 to 0.018 mm at the first molars, 0.012 to 0.017 mm at the second premolars, and 0.008 to 0.036 mm at the first premolars. Cronbach’s alpha analysis for reliability revealed a value of 0.998.

### BABT changes

**Fig. 4:**
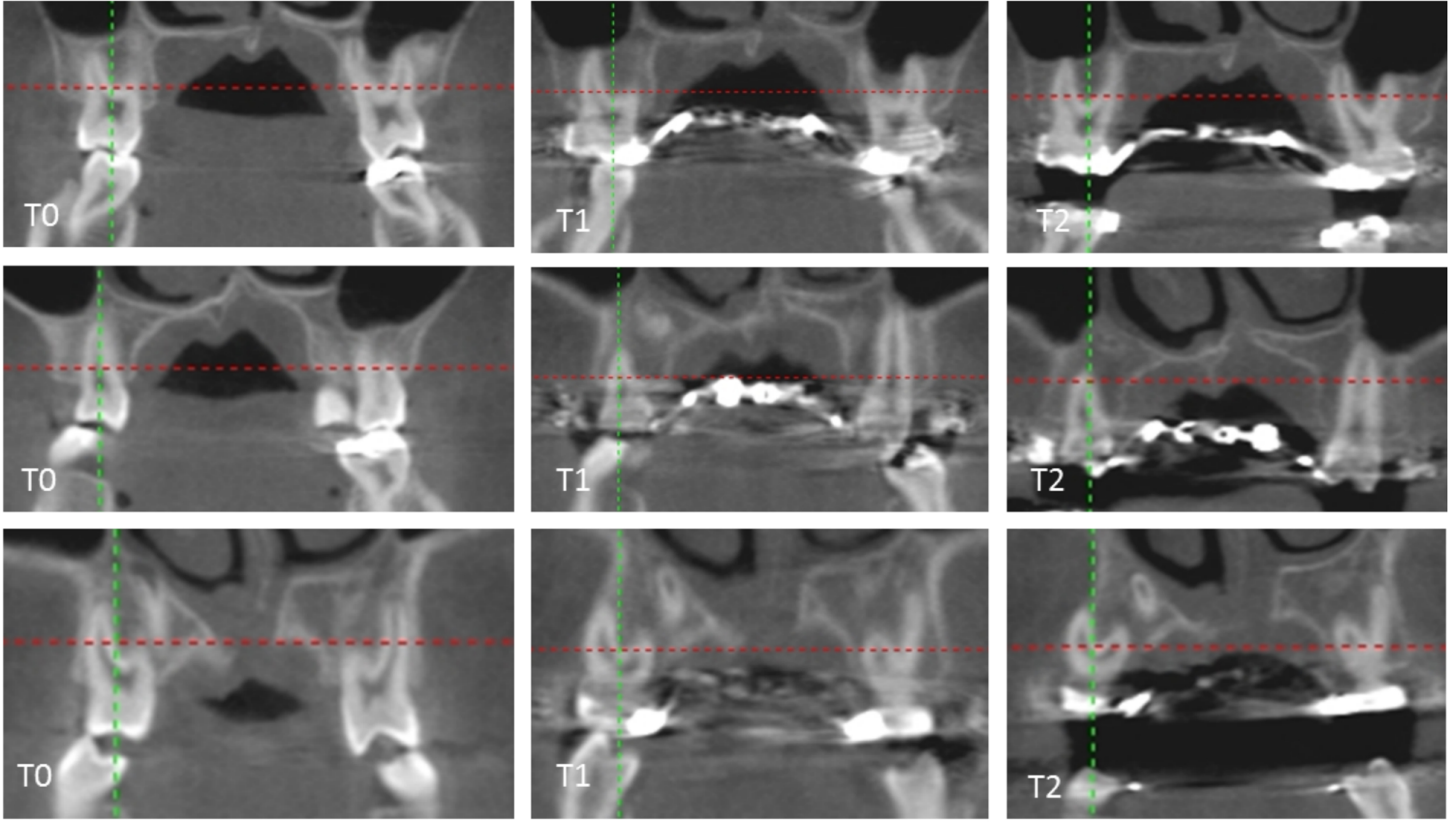
The changes of alveolar bone thickness at the first molars (upper row), second premolars (middle row), first premolars (bottom row) before expansion (T0), and 7-week Alt-RAMEC (T1). The first premolars (bottom row) had the greatest buccal alveolar bone thickness reduction at 7-week Alt-RAMEC

The 7-week Alt-RAMEC significantly (*p* < 0.05) reduced the BABT with maximum reduction at all four axial sections (AS1–4) at the first premolars (0.54 ~ 0.70 mm, *p* < 0.05), and a lesser reduction at the second premolars (AS1–4) (0.14 ~ 0.36 mm, *p* < 0.05), and the cervical region (AS1 and 2) of first molars. A non-significant reduction was noticed at the apical region (AS3 and 4) at the mesial (0.0 ~ 0.00 mm, *p* > 0.05) and distal roots (0.05 ~ 0.06 mm, *p* > 0.05) of first molars. The BABT reduction by the 7-week Alt-RAMEC was all within the scope of the initial BABT (Table 2, Fig. 4).

**Table 2:** Intra-group comparison of buccal alveolar bone thickness (BABT) measurements before expansion (T0) and after 7-week Alt-RAMEC (T1) with the paired *t* test (*p* < 0.05)

		T0 (mm)	T1 (mm)	T0–T1 (mm) *p* value
1st molar–mesial	AS1	1.56 ± 0.09	1.31 ± 0.06	− 0.25 ± 0.14(0.000*)
	AS2	1.50 ± 0.11	1.36 ± 0.12	− 0.14 ± 0.12(0.000*)
	AS3	1.56 ± 0.09	1.54 ± 0.09	−0.01 ± 0.02(0.686)
	AS4	1.56 ± 0.09	1.55 ± 0.09	−0.00 ± 0.02(0.954)
1st molar –distal	AS1	2.56 ± 0.16	2.28 ± 0.16	−0.28 ± 0.04(0.000*)
	AS2	2.53 ± 0.19	2.14 ± 0.10	−0.34 ± 0.04(0.000*)
	AS3	2.54 ± 0.19	2.54 ± 0.19	−0.05 ± 0.10(0.896)
	AS4	2.60 ± 0.19	2.54 ± 0.19	−0.06 ± 0.05(0.731)
2nd pre molar	AS1	1.28 ± 0.06	1.14 ± 0.11	−0.14 ± 0.09(0.000*)
	AS2	1.29 ± 0.05	1.08 ± 0.10	−0.20 ± 0.09(0.000*)
	AS3	1.29 ± 0.05	1.08 ± 0.10	−0.21 ± 0.10(0.000*)
	AS4	1.29 ± 0.05	0.93 ± 0.06	−0.36 ± 0.06(0.000*)
1st pre molar	AS1	0.81 ± 0.10	0.26 ± 0.05	−0.54 ± 0.11(0.000*)
	AS2	0.81 ± 0.10	0.25 ± 0.04	−0.56 ± 0.11(0.000*)
	AS3	0.93 ± 0.06	0.23 ± 0.06	−0.70 ± 0.09(0.000*)
	AS4	0.83 ± 0.11	0.25 ± 0.05	−0.57 ± 0.11(0.000*)

*Statistically significant (*p* < 0.05)

*AS1,* Axial section 1; *AS2,* Axial section 2; *AS3,* Axial section 3; *AS4,* Axial section 4

‘-‘negative values indicate a decrease

### PABT changes

The 7-week Alt-RAMEC significantly (*p* < 0.05) increased the PABT at all four axial sections (AS1–4) at the first premolars (0.31 ~ 0.41 mm, *p* < 0.05) and the cervical region (AS1 and 2) of first molars (0.32 and 0.32 mm, *p* < 0.05). A non-significant difference was noted at all fours sections of the second premolars and at the apical region (AS3 and 4) of the first molars root (Table 3).

**Table 3:** Intra-group comparison of palatal alveolar bone thickness (PABT) measurements before expansion (T0) and after 7-week Alt-RAMEC (T1) with the paired t test (*p* < 0.05)

		T0 (mm)	T1 (mm)	T0–T1 (mm) *p* value
1st molar –palatal	AS1	1.96 ± 0.27	2.29 ± 0.24	0.32 ± 0.15(0.000*)
	AS2	1.97 ± 0.27	2.30 ± 0.24	0.32 ± 0.14(0.000*)
	AS3	1.99 ± 0.25	2.05 ± 0.21	0.06 ± 0.20(0.308)
	AS4	1.99 ± 0.25	2.05 ± 0.21	0.06 ± 0.20(0.308)
2nd pre molar	AS1	2.57 ± 0.23	2.57 ± 0.22	0.00 ± 0.05(0.943)
	AS2	2.61 ± 0.24	2.61 ± 0.24	0.00 ± 0.04(0.988)
	AS3	2.61 ± 0.24	2.60 ± 0.20	0.00 ± 0.08(0.889)
	AS4	2.61 ± 0.24	2.62 ± 0.24	0.01 ± 0.08(0.923)
1st pre molar	AS1	2.53 ± 0.25	2.91 ± 0.29	0.41 ± 0.28(0.000*)
	AS2	2.58 ± 0.26	2.92 ± 0.29	0.34 ± 0.29(0.000*)
	AS3	2.60 ± 0.28	2.93 ± 0.30	0.33 ± 0.30(0.001*)
	AS4	2.63 ± 0.29	2.94 ± 0.32	0.31 ± 0.30 (0.001*)

*Statistically significant (*p* < 0.05)

*AS1,* Axial section1; *AS2,* Axial section 2; *AS3,* Axial section 3; *AS4,* Axial section 4

## Discussion

Previous CT studies of the effects of RME on palatal and buccal alveoli evaluated only on one axial section [12, 14–16], which might not sufficiently delineate the effects of RME on the entire alveolar bone thickness. This present study could be the first to investigate the immediate effects of Alt-RAMEC on the alveolar bone of the same patients by evaluating on four CBCT axial sections. Our results revealed that the Alt-RAMEC reduced the BABT (0.54 ~ 0.70 mm) and thickened the PABT (0.31 ~ 0.41 mm) at the anterior anchor teeth. All the BABT reduction by the Alt-RAMEC was within the scope of the initial BABT, which indicated that the Alt-RAMEC did not cause root or bony dehiscence or compromise the periodontal support of all the anchor teeth.

The first premolars had the greatest BABT reduction by the Alt-RAMEC. This could be due to the double-hinged expander which expands maxilla more at the anterior than posterior [5, 6], and the first premolars were the anterior anchor teeth. The first premolars therefore were expanded in a wider range and heavier force resulting in a more BABT reduction. The first premolars also had the thinnest initial BABT, and a thinner initial BABT has been revealed having more buccal bone reduction and deleterious post-expansion effects [15, 18]. The range of expansion and initial BABT of the anchor teeth of a maxillary expander seems critical to the BABT reduction no matter if it is RME or Alt-RAMEC. On the other hand, the second premolars had the least and negligible BABT reduction because they were not banded or bonded to the expander, and they were the indirect anchor teeth.

Maxillary expansion results in either buccal crown tipping of the anchor teeth of a hyrax expander [21, 22] or bodily movement of the anchor teeth of a Hass type expander with palatal acrylic pads [12, 17, 21, 22]. Interestingly, the double-hinged expander resulted in buccal crown tipping of the posterior anchor teeth but bodily movement of the anterior anchor teeth. Our results revealed that the BABT reduction and PABT thickening were both more at the cervical (AS1 and 2) than at the apical regions (AS3 and 4) of the posterior anchor teeth (fist molars), but they were similar at the cervical and apical regions of the anterior anchor teeth (first premolars).

In comparison to the 1-week RME protocols [15, 16, 18], the protocol of Alt-RAMEC had three more sets of alternating rapid expansions and constrictions of maxilla. And it is reasonable to assume that the Alt-RAMEC has more impacts on the BABT reduction and PABT thickening than RME and has more root and alveolar bone dehiscence. However, the results of this study showed that the BABT reduction and PABT thickening were both within the scope of initial BABT and PABT. The impacts of Alt-RAMEC were less than we expected.

This could be due to the alternating expansion and constriction that is a reciprocal recovery period to each other, which makes the impacts on alveolus less. For a 7-week Alt-RAMEC, the three constrictions reciprocally relieve the expansion force and alternate the buccal alveolus from pressure to tension side and the palatal alveolus from tension to pressure side. Similarly, the four expansions reciprocally relieve the constriction force and alternate the buccal alveolus from tension back to pressure side and the palatal alveolus from pressure back to tension side. At the same time, the expansion and constriction force also reciprocally disarticulates the circumaxillary sutures with less impact on the sutures. It has been suggested that maxillary orthopedic protraction is better before fusion of the circumaxillary sutures [2, 5, 23, 24].

There are certain limitations of this clinical study. Although, the ICC was .992–.996 indicating high reliability, however, the validity (reproducibility) of this study would be more meaningful if the study’s measurement was conducted by at least two individuals with an inter-operator reliability testing. This study was a case-series cohort study rather than a randomized clinical trial. Our findings on BABT reduction and PABT thickening were the immediate effects observed after 7 weeks of the Alt-RAMEC protocol without removal of the expander. The consequence or recovery process of the BABT reduction and PABT thickening by Alt-RAMEC were not studied. It has been revealed that the BABT reduction by RME was reversible and had no evident deleterious effects and would recover after 6 months of retention period [12, 25].The current study did not investigate the impacts of Alt-RAMEC on alveolar bone height of the anchor teeth of expander. It has been revealed that RME seldom causes alveolar bone apical displacement, especially when the process is sterile and noninflammatory [14]. The overall impacts of Alt-RAMEC on the alveolar bone still need further studies.

## Conclusions

A 7-week protocol of Alt-RAMEC with double-hinged expander for maxillary protraction might reduce the buccal alveolar bone thickness of the expander’s anchor teeth, although the reduction is within the scope of initial alveolar thickness of the expander’s anchor teeth.

## References

[1] Isci D, Turk T, Elekdag-Turk S (2010). Activation–deactivation rapid palatal expansion and reverse headgear in class III cases. Eur J Orthod.

[2] Liou EJW, Chen PKT (2003). New orthodontic and orthopaedic managements on the premaxillary deformities in patients with bilateral cleft before alveolar bone grafting. Surg Pract.

[3] Liou EJ-W, Tsai W-C (2005). A new protocol for maxillary protraction in cleft patients: repetitive weekly protocol of alternate rapid maxillary expansions and constrictions. Cleft Palate Craniofac J.

[4] Liou E (2005). Toothborne orthopedic maxillary protraction in class III patients. J Clin Orthod.

[5] Liou E (2005). Effective maxillary orthopedic protraction for growing class III patients: a clinical application simulates distraction osteogenesis. Prog Orthod.

[6] Wang Y-C, Chang PM, Liou EJ-W (2009). Opening of circumaxillary sutures by alternate rapid maxillary expansions and constrictions. Angle Orthod.

[7] Tagawa DT, Bertoni CLSC, MAE M, Redivo Junior M, LAdA A (2012). Orthopedic treatment of class III malocclusion with rapid maxillary expansion combined with a face mask: a cephalometric assessment of craniofacial growth patterns. Dental Press J Orthod.

[8] Williams MD, Sarver DM, Sadowsky PL, Bradley E (1997). Combined rapid maxillary expansion and protraction facemask in the treatment of class III malocclusions in growing children: a prospective long-term study. Semin Orthod.

[9] Vaughn GA, Mason B, Moon H-B, Turley PK (2005). The effects of maxillary protraction therapy with or without rapid palatal expansion: a prospective, randomized clinical trial. Am J Orthod Dentofac Orthop.

[10] Foersch M, Jacobs C, Wriedt S, Hechtner M, Wehrbein H (2015). Effectiveness of maxillary protraction using facemask with or without maxillary expansion: a systematic review and meta-analysis. Clin Oral Investig.

[11] Macdonald KE, Kapust AJ, Turley PK (1999). Cephalometric changes after the correction of class III malocclusion with maxillary expansion/facemask therapy. Am J Orthod Dentofac Orthop.

[12] Ballanti F, Lione R, Fanucci E, Franchi L, Baccetti T, Cozza P (2009). Immediate and post-retention effects of rapid maxillary expansion investigated by computed tomography in growing patients. Angle Orthod.

[13] Fuhrmann R, Bücker A, Diedrich P (1995). Assessment of alveolar bone loss with high resolution computed tomography. J Periodontal Res.

[14] Garib DG, Henriques JFC, Janson G, de Freitas MR, Fernandes AY (2006). Periodontal effects of rapid maxillary expansion with tooth-tissue-borne and tooth-borne expanders: a computed tomography evaluation. Am J Orthod Dentofac Orthop.

[15] Garib DG, Henriques JFC, Janson G, Freitas MR, Coelho RA (2005). Rapid maxillary expansion—tooth tissue-borne versus tooth-borne expanders: a computed tomography evaluation of dentoskeletal effects. Angle Orthod.

[16] Podesser B, Williams S, Crismani AG, Bantleon H-P (2007). Evaluation of the effects of rapid maxillary expansion in growing children using computer tomography scanning: a pilot study. Eur J Orthod.

[17] Starnbach H, Bayne D, Cleall J, Subtelny JD (1966). Facioskeletal and dental changes resulting from rapid maxillary expansion. Angle Orthod.

[18] Rungcharassaeng K, Caruso JM, Kan JY, Kim J, Taylor G (2007). Factors affecting buccal bone changes of maxillary posterior teeth after rapid maxillary expansion. Am J Orthod Dentofacial Orthop.

[19] Basili C, Costa H, Sasaguri K, Akimoto S, Slavicek R, Sato S (2009). Comparison of the position of the mandibular fossa using 3D CBCT in different skeletal frames in human caucasic skulls. Int J Stomatol Occlusion Med.

[20] Houston W (1983). The analysis of errors in orthodontic measurements. Am J Orthod.

[21] Adkins MD, Nanda RS, Currier GF (1990). Arch perimeter changes on rapid palatal expansion. Am J Orthod Dentofac Orthop.

[22] Bishara SE, Staley RN (1987). Maxillary expansion: clinical implications. Am J Orthod Dentofac Orthop.

[23] Baccetti T, Franchi L, McNamara JA (2005). The cervical vertebral maturation (CVM) method for the assessment of optimal treatment timing in dentofacial orthopedics. Semin Orthod.

[24] Yen SL-K (2011). Protocols for late maxillary protraction in cleft lip and palate patients at childrens hospital. Los Angeles Semin Orthod.

[25] Akyalcin S, Schaefer JS, English JD, Stephens CR, Winkelmann S (2013). A cone-beam computed tomography evaluation of buccal bone thickness following maxillary expansion. Imaging Sci Dent.

